# Outcomes of Spinal Manipulation and Neurological Rehabilitation for a Patient With Chronic Tension-Type Headaches and a Prior History of Head Trauma

**DOI:** 10.7759/cureus.91838

**Published:** 2025-09-08

**Authors:** Daniel J Perman, Shelbi Hughes, Paula Murillo, Krista Ward

**Affiliations:** 1 Nexus Brain Center, Life Chiropractic College West, Hayward, USA; 2 Health Center, Life Chiropractic College West, Hayward, USA; 3 Research Department, Life Chiropractic College West, Hayward, USA

**Keywords:** brain concussion, chronic tension-type headache (ctth), neurological rehabilitation, single-case study, spinal manipulation

## Abstract

Chronic tension-type headaches (TTH) can be a debilitating condition that may include peripheral vestibular system involvement. This case report describes the response to spinal manipulation (SM) and neurologic rehabilitation for a 44-year-old male with TTH complicated by a history of concussion. The patient presented to a chiropractic teaching clinic with over 15 years of daily headaches, lasting about seven hours, and rated 5/10 on a visual analog scale. The patient recalled no single causal event. Several years prior to headache onset, the patient had multiple concussions from football and military/police force occupation. Baseline findings included nystagmus, unsteady gaze holding, and latency and fatigue with saccades in all directions. Over the course of seven months, the patient received 24 visits of SM and 17 treatments of trans-auricular vagus nerve stimulation, repetitive peripheral somatosensory stimulation, photobiomodulation, and gaze stabilization exercises. The patient reported 50% headache improvement after seven weeks and 90% improvement at 20 weeks, at which point headache frequency was once a week for one hour or less. No nystagmus was observed in the 27-week exam. Chronic TTH improved with SM and neurologic rehabilitation. While past clinical trials document similar TTH improvement with SM, little is known about neurological rehabilitation treatments for TTH. This case study suggests that research in this area may be warranted.

## Introduction

Tension-type headaches (TTH) affect 30-78% of the population, and chronic TTH is considered to be a debilitating disease that greatly affects quality of life (QoL) and contributes to significant disability and costs [1,2]. Diagnostic criteria state that chronic TTH headaches (CTTH) must occur at least 15 days per month and at least 180 days or more per year [1]. The headaches must also last hours to days and have at least two of the following criteria: bilateral location, pressing or tightening quality, mild-to-moderate intensity, and not affected negatively by physical activity [1]. Patients with TTH may also present with peripheral vestibular system involvement. In one study, 3% of TTH patients exhibited spontaneous nystagmus, and 12% presented with bilateral vestibulopathy [3].

Previous research suggests spinal manipulation therapy (SMT) can help relieve TTH [4-6]. In one study of eight sessions of upper cervical SMT and patient exercise, TTH frequency was reduced by 3.3 days and headache pain by 3.8 on the visual analog scale (VAS) [4]. The decreased frequency and pain intensity persisted three months post intervention and were greater than the decreases observed for participants receiving exercise only or exercise and myofascial release [4]. Little is known about the clinical effectiveness of neurorehabilitation therapies to address TTH.

This case report describes the response to neurological rehabilitation therapies and SMT for a patient with CTTH complicated by a history of multiple concussions and post-traumatic stress disorder (PTSD). This retrospective case report received a determination of not human subjects research. The patient consented to publication. The authors used the Consensus-based Standards for the Reporting of Articles in Healthcare (CARE) checklist as a writing framework.

## Case presentation

Patient history and exam

A 44-year-old male patient presented to a chiropractic school’s health center (HC) with chronic moderate intermittent cephalgia, which began in his mid-late 20s. He described the pain as sharp/stabbing in the temporal region and throbbing/pounding in the occipital region. VAS pain was reported as 5/10. The headaches occurred daily, beginning in the mornings and lasting about seven hours. He reported the pain was exacerbated by wearing a helmet for work and looking down for long periods of time, but was not aggravated by physical activity. The headache pain was relieved or reduced by massage and over-the-counter medication. The patient recalled no event that caused the headaches; they came on slowly in frequency and severity until they became continuous and unbearable. The patient did not report any prodrome symptoms, although sometimes he saw kaleidoscope patterns in his visual field when temporal headaches were bad. Additional complaints included shoulder pain, low back pain, dizziness, bilateral tinnitus, and poor short-term memory.

The patient denied a family history of chronic disease. Medications included bupropion for anxiety and depression, which the patient began two weeks prior to initiating SMT; amlodipine for hypertension; naproxen for inflammation; and Symbicort for asthma. Past history included five mild traumatic brain injuries (mTBIs) due to blunt sport and occupational traumas during the patient’s early 20s. The only medical treatments the patient received for these were jaw and dental work. The patient may have also experienced mTBIs due to military blast trauma. Two years prior to initiating care, the patient was diagnosed at the United States Department of Veterans Affairs for PTSD and temporomandibular joint dysfunction.

The patient reported contentment with his police officer job, 50-60 hours per week. He reported enjoying exercising three to five days/week for one to two hours. Alcohol use was reported as one to three beverages per week, and caffeine was reported as three cups per day. The patient denied tobacco use. The patient described getting about five hours of interrupted sleep/night. Health-related quality of life (HRQoL) was measured with the RAND-36 instrument, which is equivalent to the commonly used SF-36 survey [7]. RAND-36 scores are displayed in Table 1. 

**Table 1:** Patient-assessed outcomes for the initial health center (HC) exam and week 7 and week 20 follow-up HC exams. The RAND-36 Health Survey comprises 36 questions that are scored within eight different health domains and a question about how health in general compares to one year prior [7].

Variable	Initial exam	Week 7 exam	Week 20 exam
Headache Frequency	Daily	2-3 times/week	1 time/week
Headache Duration	7 hours	1-2 hours	1 hr or less
Percent Headache Improvement	-	50%	90%
General State of Well-Being (0-10)	8	7	7
General Outlook and Attitude (0-10)	8	8	8
Stress	7	5	6
Past 30 Days, Healthy/Full of Energy	15	20	25
Past 30 Days, Physical Health Not Good	10	3	5
Sleep Duration	5 hrs	3-5 hrs	5-6 hours
RAND-36 Domain Scores			
Physical functioning	85	90	95
Role limitations: physical health	0	50	100
Role limitations: emotional	67	100	33
Energy/fatigue	60	55	55
Emotional well-being	68	68	68
Social functioning	88	50	63
Bodily pain	45	58	78
General health	75	80	80
RAND-36 Health Change Scores	50	75	100

**Figure 1:**
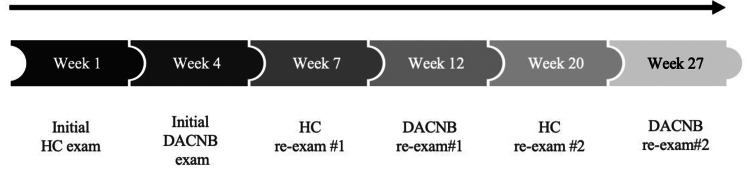
Assessment timeline for patients receiving spinal manipulation in the chiropractic teaching clinic (HC) and neurologic rehabilitation therapies by a Diplomate of the American Chiropractic Neurology Board (DACNB).

The patient was initially examined in the HC, and then he was referred to on-site neurology assessment and rehabilitation care by a Diplomate of the American Chiropractic Neurology Board (DACNB). In the course of seven months, the patient was re-examined twice in the HC (weeks 7 and 20) and twice by the DACNB (weeks 12 and 27; Figure 1).

At the time of the patient’s initial HC exam, he had a body mass index of 30.3. Blood pressure was 135/82, and heart rate and respiration were within normal limits. The headache symptoms were not reproduced with the cervical orthopedic exam. Neurologic findings were normal, except for decreased vibration sensation along the right T1 dermatome. During the cranial nerve exam, the patient was asked to fixate on a point on a distant wall. He was then asked to direct his vision to a business card held about 12 inches from his face. The patient reported blurry words for 10-15 seconds before his eyes were able to accommodate. See Table 1 for additional exam findings.

Cervical X-rays were ordered due to trauma history and spinal tenderness with palpation. X-ray findings included right convex lower cervical scoliosis; flattening of the cervical lordosis; uncovertebral hypertrophy C5-C6 on the left; and facet arthrosis C4-C7 on the left.

**Figure 2:**
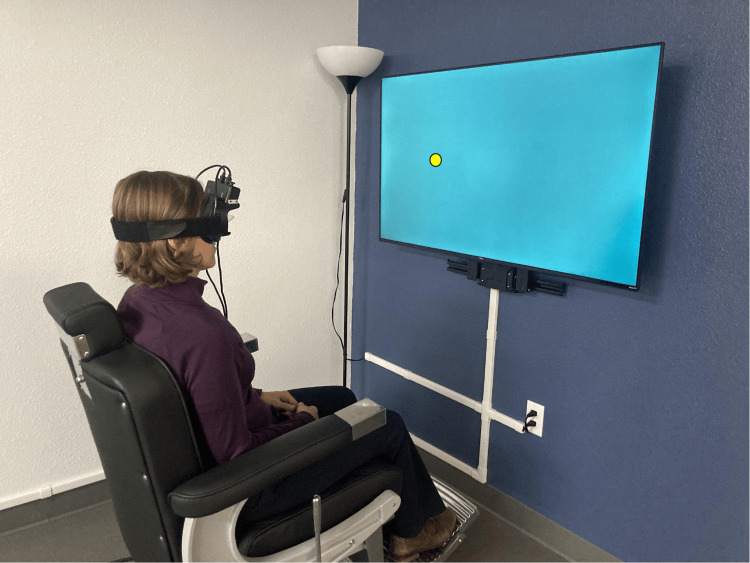
Video-oculography (VOG) setup. The image is from a research staff member, not the patient. The yellow target is enlarged for image visualization.

Initial DACNB exam included video-oculography (VOG) assessments of spontaneous nystagmus, gaze holding, pursuits, saccades, and optokinetic nystagmus using a Micromedical Visual Eyes 525 device (Interacoustics, Middelfart, Denmark). All targets were positioned 36 inches from the patient on a 50-inch LCD TCK 59S455 television (Figure 2). A dot on the screen was centered relative to the patient's eyeline. The patient was instructed to hold their head still and open their eyes wide while staring at a series of targets.

The DACNB exam also included an assessment of proprioceptive, vestibulospinal, and oculospinal function using the Comprehensive Assessment of Postural Systems (CAPS®) (Vestibular Technologies LLC, Cheyenne, WY). The CAPS assessment included two protocols for assessing balance, including the Modified Clinical Test for Sensory Interaction on Balance (mCTSIB), a commonly used test with the addition of head movements as described by Cohen et al. [8]. See Table 2 for initial VOG, CAPS, and other bedside neurologic exam findings.

**Table 2:** Assessments of a Diplomate of the American Chiropractic Neurology Board (DACNB).

Exam Procedure Details	Baseline DACNB exam (Week 4)	DACNB first re-exam (Week 12)	DACNB second re-exam (Week 27)
Video-oculography (VOG) Testing Spontaneous Nystagmus with fixation removed: Performed over 15 seconds	Mild convergence myospasm was observed when fixation was eliminated.	Mild left beating nystagmus was observed when fixation was eliminated.	No nystagmus or convergence myospasm was observed when fixation was eliminated.
VOG Testing Gaze Holding: 5 directions (Center, 30 degrees to the right, 30 degrees to the left, 18 degrees upwards, 18 degrees downwards) over 15 seconds each	Gaze holding was unsteady in all directions.	Gaze holding was normal.	Gaze holding was unsteady to the right.
VOG Testing Pursuits: Tested horizontally at 20 degrees and vertically at 18 degrees of motion and speeds ranging from 0.1 to 0.5 Hz horizontally and 0.1 to 0.3 Hz vertically.	Fatigue in all directions.	Mild horizontal oscillations with upward pursuits.	Mild horizontal oscillations with upward pursuits.
VOG Testing Saccades: Tested horizontal and vertical separately over 45 seconds each (Amplitude of saccades was random, but was between -20,+20 degrees for horizontal saccades left and right, and -15, +15 degrees downwards and upwards).	Latency of the right eye looking left. Fatigue with saccades in all directions. See Figure 3 for baseline horizontal saccade report.	Saccade latency and velocity were within normal limits for both eyes in both directions. See Figure 4 for the re-exam 1 horizontal saccade report.	Saccade latency and velocity were within normal limits for both eyes in both directions.
Optokinetic (OPK) Testing: Optokinetic stimulation was performed in all directions, right, left, up, and down. A pattern of white and yellow circles on a red background was placed on the screen. For 15 seconds, the balls moved in a singular direction. The circles for vertical testing were smaller than horizontal testing. Right and left OPKs were tested at 30 degrees/sec each over 15 seconds, and up and down OPKs were tested at 20 degrees per second over 15 seconds.	Marked Fatigue with all OPKs.	Slight fatigue with leftward OPKs. Dysmetria with upward OPKs.	Mild dysmetria with upward and downward OPKs.
Comprehensive Assessment of Postural Systems (CAPS) Assessment: Patient is advised to stand on a force plate as still as possible. The device measures sway to determine the patient's stability during 30-second-long trials. Trials include standing on and off a foam pad, with eyes open and closed, and with their head placed in different positions while standing on a foam pad with their eyes closed.	Increased sway with cervical extension and with left cervical rotation when standing on a foam pad with eyes closed.	No notable deficits.	Increased sway with cervical extension when standing on a foam pad with eyes closed.
Romberg's Test: Patient asked to stand with feet touching, shoes off, and arms to the side with eyes open and then eyes closed.	Leftward initial sway with a right/posterior sustained sway pattern.	No notable initial sway with a posterior sustained sway pattern.	No notable initial sway with a posterior sustained sway pattern.
Perturbed Romberg's: Patient asked to stand on a foam pad with feet touching, shoes off, and arms to the side with eyes open and then eyes closed.	Leftward initial sway with a rightward and an anterior sustained sway pattern.	Leftward initial sway with right-to-left sustained sway pattern.	Rightward initial sway with a rightward sustained sway pattern.
1 Leg Stand: Patient asked to stand on one leg (right and left) for 10 seconds with eyes closed	Decreased stability when standing on the left leg.	Decreased stability when standing on the right leg.	Decreased stability when standing on the right leg.

Diagnosis

The patient’s history and presentation met all of the International Classification of Headache Disorders (ICHD) criteria for intractable CTTH [1]. A lack of temporal relationship between the initiation of the headaches and either a cervical disorder or prior concussion differentiated the diagnosis from cervicogenic headaches and persistent headaches attributed to traumatic injury. The patient had no nausea, vomiting, photophobia, or phonophobia, which excluded migraine headaches. There were no financial or cultural diagnostic challenges. The patient was also diagnosed with unspecified subjective visual disturbances.

Treatment

The patient received SMT approximately two times a week in the HC for the first seven weeks of care (Figure 1). Between weeks seven and 19, the frequency of SMT decreased to once per week, and then after 20 weeks, to two times per month. Between week four and week 27 of care, the patient was also seen 17 times by the DACNB.

Treatment by the DACNB was delayed until week four due to scheduling and referral lag. DACNB visits included the following: trans-auricular vagus nerve stimulation using high volt galvanic stimulator CS6102 (Medical Systems, Minneapolis, MN) (15 minutes, alternating sides between sessions); repetitive peripheral somatosensory stimulation of the right trigeminal nerve (three sets of 15 seconds at three locations with the Cadwell Sierra Wave instrument (Cadwell Industries, Kennewick, WA); cold laser therapy; and transcranial photobiomodulation (six minutes) with the FX405 laser (Erchonia, Fountain Inn, SC). Using Dynavision D2TM (Dynavision, Brecon, OH), the patient also received training (10-15 minutes/visit) in spatial awareness, reaction time, and cognitive and hand-eye coordination with progressively more challenging exercises each visit.

The patient was also instructed in convergence and horizontal and vertical gaze stability exercises to be performed three times/day. Convergence exercises involved moving a popsicle stick with a target at the tip from arm's distance towards the nose while trying to keep the target in focus. Gaze stability exercises were adapted from Carrick et al.’s work [9]. The patient was compliant with exercises (two times/day) and tolerated treatments well. No adverse events were observed.

Outcomes

The patient initially presented to the clinic with headaches occurring every day for about seven hours a day, with a 5/10 VAS for pain. After five months of care, the patient reported 90% headache improvement with frequency decreasing from seven days/week to about once a week and duration decreasing from seven hours/headache day to one to two hours/headache day. Between the initial and 20-week exams, the patient reported a 67% increase in the number of past 30 days feeling healthy and full of energy, and a 50% decrease in the past 30 days when physical health was not good. QoL improved for five of the nine RAND-36 subscales (Table 1). While the patient did not report an increase in hours of sleep per night, he did describe less difficulty falling asleep following care.

**Figure 3:**
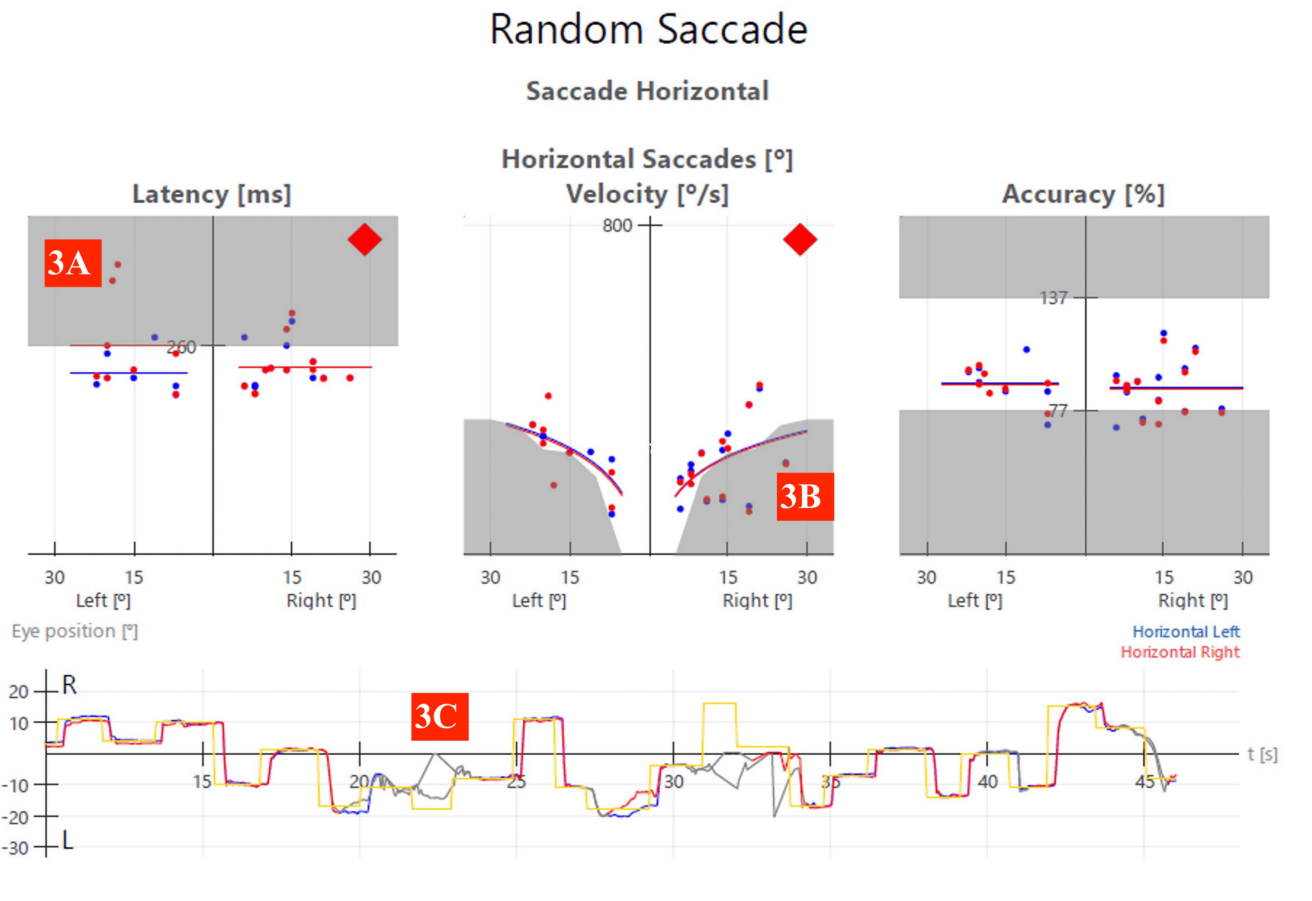
Video-oculography (VOG) test report for the horizontal saccade baseline neurology exam (week four). Data points in the grey zones are outside normal ranges. Latency is outside the normative values for the right eye looking left (Figure 3A). Velocity was outside the normative values for both eyes looking right (Figure 3B). The patient’s eyes also closed several times during the test when the patient appeared to briefly fall asleep as indicated by the grey lines in the bottom of the saccade report (Figure 3C) and in Appendix Video 1.

**Figure 4:**
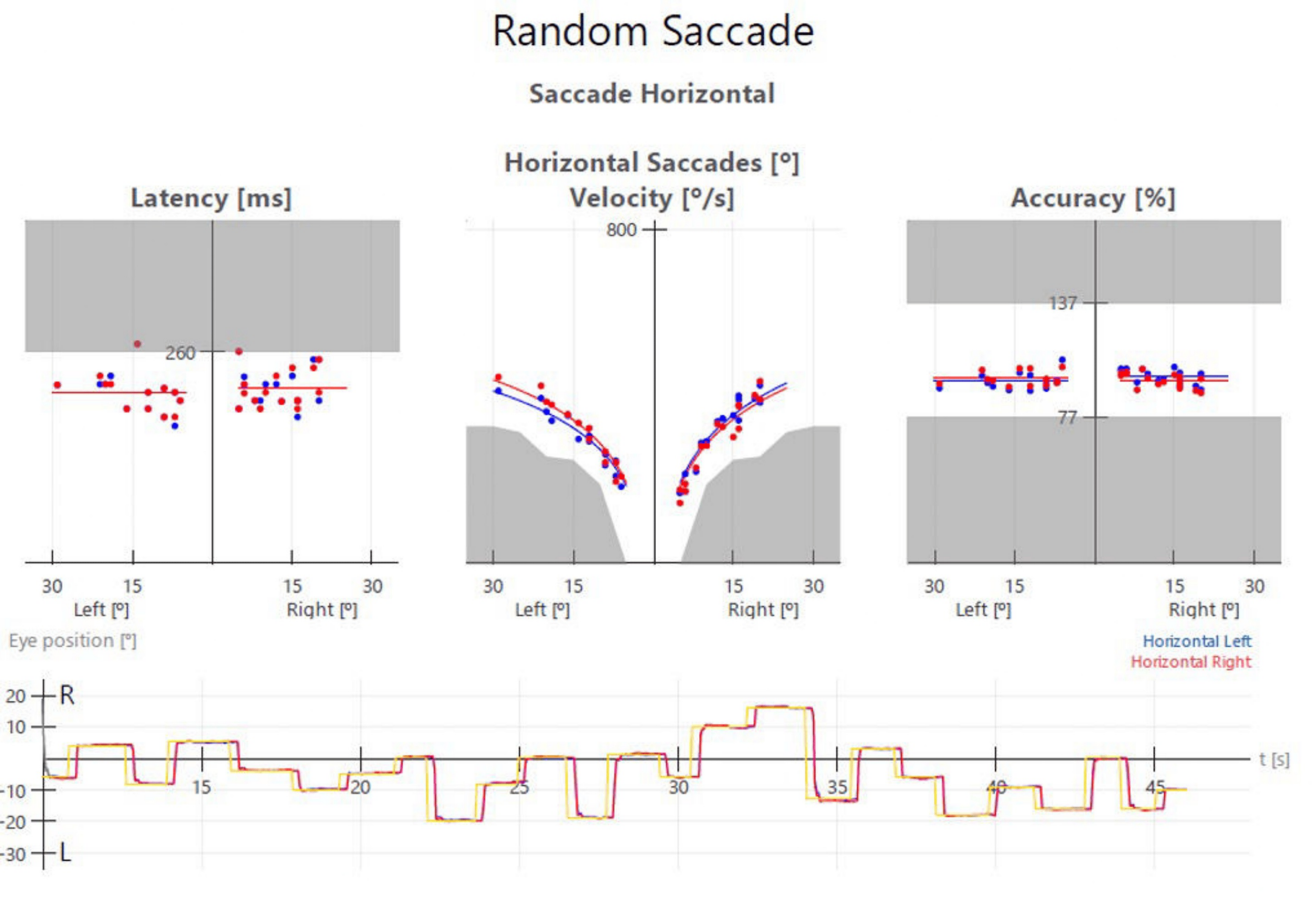
Video-oculography (VOG) test report for the horizontal saccade neurology re-exam #1 (week 12). Data points in the grey zones are outside normal ranges. Saccade latency and velocity were within normal limits for both eyes in both directions, and the patient’s eyes did not fatigue (see also Appendix Video 2).

Clinician-reported outcomes also improved. Functional oscillopsia was not seen after the initial exam, and mild to no nystagmus was seen in the first and second DACNB re-exams, respectively (Table 2). In addition, saccade latency was reduced, and velocity was improved from the initial exam to the first DACNB re-exam (Figures 3-4 and Appendix Videos 1-2).

## Discussion

The prevalence of TTH and PTSD in the veteran population is estimated to be 12% and 40%, respectively, and veterans with PTSD have four times the odds of migraine or TTH [10]. Although the term “tension type” may connotate that the headache is due to stress and muscular tension, previous authors have suggested these headaches have multiple concurrent mechanisms of pathophysiological origin with more of a neurological basis than any psychological origin [11,12]. The etiology of CTTH includes increased sensitization at the neurological level due to continued and chronic pain signals coming from the myofascial tissues surrounding the head [13]. This case exemplifies a multifactorial etiology, given the patient’s history of PTSD and multiple head injuries.

Prior research suggests that the patient’s presentation of headaches and neuro-ocular findings is not unique, especially given his history of mTBIs. Patients with mTBIs have presented with accommodative dysfunction and saccadic latency and fatigue, even several years post concussion [14]. Blast exposures can injure the frontal and prefrontal cortices and cerebellum associated with ocular motor control [14].

This case study describes the improvement of CTTH, QoL, and neurologic findings during seven months of SMT and neurologic rehabilitation therapies. Strengths of this study are the use of validated patient and clinician-reported outcome measures. The patient experienced reduced headache frequency and function; improved balance with Romberg’s test; a reduction in nystagmus and latency/fatigue with saccades with VOG assessments; and reduced limitations due to physical health as reported on the RAND-36. These results are similar to previously reported reductions in TTH frequency and photophobia with suboccipital soft tissue inhibition and occiput-atlas-axis manipulation [6]. Neuromodulation is a recommended therapy for migraines [15], and this case supports investigating its use in CTTH.

Due to the inherent limitations of case reports, the results of this study can not be generalized nor used as evidence for the efficacy of the treatments described. In addition, this retrospective observation prevents identification of the specific origins of the patient’s symptoms, and the exact causes of his symptomatic relief remain unknown. Specifically, we do not know if the patient’s presentation of headaches and neuro-ocular findings were related to prior mTBIs or if the improvements in balance and eye tracking were due to SMT, neurologic rehabilitation, and/or another contributing factor. The patient may have experienced changes in his health due to the bupropion he started taking two weeks prior to initiating SMT. While the authors of this case study found no studies on bupropion or other atypical antidepressants as a therapy for headaches, there is evidence for other antidepressants having a therapeutic effect on CTTH [16]. The patient also received myofascial release for right shoulder pain during the treatment phase, which may have also impacted his QoL.

Future randomized controlled clinical studies are needed to assess the efficacy of neurologic rehabilitation for CTTHs. To better document the clinical significance and to calculate the reliable change index for future related case studies, further research is needed to establish the minimal clinically important difference and standard errors of measurement for the RAND-36 in headache populations, similar to the Swedish study published by Grönkvist et al. in 2024 [17].

## Conclusions

This case study describes the response to SMT and neurological rehabilitation for CTTH in a 44-year-old male patient. The patient's QoL and headache frequency and intensity improved, and clinical outcomes included reduced oscillopsia, nystagmus, and saccade latency. The results can not be generalized from this single patient. Future research is needed to identify the clinical significance of these outcomes and to test the efficacy of the treatments in a large sample with a randomized controlled clinical trial design.

Meanwhile, the results of this study and the cited literature in the report support thorough neuro-ocular assessments in patients with CTTH, especially those with a history of concussion. SMT may provide benefits for post concussive patients with CTTH, a finding supported by previous research on SMT and TTH. Furthermore, this case study indicates that neuro-rehabilitation therapies may prove beneficial in patients exhibiting visual disturbances with TTH.
